# Class of 2020 in Poland: Students’ Mental Health during the COVID-19 Outbreak in an Academic Setting

**DOI:** 10.3390/ijerph18062884

**Published:** 2021-03-11

**Authors:** Tomasz Wieczorek, Agata Kołodziejczyk, Marta Ciułkowicz, Julian Maciaszek, Błażej Misiak, Joanna Rymaszewska, Dorota Szcześniak

**Affiliations:** Department and Clinic of Psychiatry, Wrocław Medical University, ul. Pasteura 10, 50-367 Wrocław, Poland; agata.kolodziejczyk@student.umed.wroc.pl (A.K.); marta.ciulkowicz@student.umed.wroc.pl (M.C.); Julian.maciaszek@umed.wroc.pl (J.M.); blazej.misiak@umed.wroc.pl (B.M.); joanna.rymaszewska@umed.wroc.pl (J.R.); dorota.szczesniak@umed.wroc.pl (D.S.)

**Keywords:** COVID-19, students, mental health, psychopathology, distress, online learning

## Abstract

The COVID-19 pandemic had led universities to introduce lockdowns, which has led to significant shifts in students’ lives. Classes were moved online, students had to leave dorms and move; they had to forgo regular meetings with their peers. Subsequently, a vital demand for examining students’ mental health emerged. The data were collected at a time when universities in Poland were under lockdowns. Participants represented students of many different fields of study. The General Health Questionnaire (GHQ-28) was used. Student’s subjective evaluation of online learning and their adaptation to academic life shifts were reviewed. A total of 1123 participants took part in this study. Relationship analysis included tests, such as U Mann–Whitney, appropriate for specific variables. The impact of variables connected with the pandemic on the GHQ scores was tested using multivariate regression analysis. The results were considered significant at a *p*-value set at 0.05. Overall, 76.96% of the participants manifested psychopathological symptoms measured by the GHQ. Four demographical variables were possibly associated with the GHQ scores: female sex, living in a big city, necessity to move back home, and being in a relationship. Negative correlations between subjective evaluation of variables concerning e-learning, such as its efficiency or quality, and the GHQ scores were found. Some variables were found to be potential protective factors, whereas others could have contributed to worsened mental health. The study provides data on students’ worsened mental health due to the COVID-19 pandemic and the shifts in academic life it caused. Therefore, recommendations for early psychosocial interventions among students are strongly advised.

## 1. Introduction

Since the discovery of the novel coronavirus COVID-19 in December of 2019 and the World Health Organization’s subsequent proclamation of a pandemic around the world in March 2020, it has become clear that the aftermath of this epidemic will impact many individuals as well as specific groups. One of the most affected group around the world has proved to be university students [1,2].

Due to the COVID-19 pandemic, policy makers introduced restrictions such as: quarantine, social distancing, obligatory mask-wearing, and strict disinfection regimes. These rules applied to individuals in almost every population. Nonetheless, specific restrictions needed to be denoted when it came to academic life. Many universities around the world, including Polish ones [3,4], have introduced lockdowns and moved curricula online [5,6]. These newly introduced regulations caused unprecedented, rapid, and unexpected shifts in students’ lives. E-learning combined with dorm-closing dispositions often forced students to move back in with their families. For many students, this meant leaving the campus and traveling back to their original place of residence, which often tended to be far from the city they were studying, often immensely affecting their studying environment. The necessity to move as well as a rapidly worsening economic situation led to job losses among students. Extra-curricular activities were postponed or cancelled, and practical teaching and internships were no longer possible [7,8], leaving students with aggravated psychosocial well-being [9]. What makes these changes even more pressing is that the time of studying is particularly important when taking into account the stage of life of young people, which has a great impact on shaping their future relationships, their sense of effectiveness, and consolidation of independence.

According to available research, the mentioned effects of closing universities resulted in negative psychological consequences among students [1,10]. Thus far, the most commonly studied group is medical students [1,9,11], whose field of study posed unique questions concerning their contact with patients, knowledge they have, and their role in containing the pandemic. Subsequently, available data on medical students’ psychosocial well-being proved them to be negatively affected by the pandemic, often manifesting psychological distress [1,8,12]. Nonetheless, whereas medical students did in fact react to the COVID-19-related changes in a unique way, due to the knowledge and responsibilities they carry as well as specific type of classes they need to attend, it is not only them who suffered and reacted to these unprecedented shifts in academic life [11,12]. These changes have influenced students around the world of many faculties and fields of study. Thus far, it has been observed that negative effects on students’ psychological well-being arise from the tiresome adjustment process to online learning [1], a lack of peer interaction resulting in feelings of loneliness [11], and uncertainty about the future, graduation, or quality of curricula [1]. It has already been denoted that those effects may lead to psychopathological symptoms and behaviors such as anxiety, depression [9], burnout, fatigue [13,14], psychotic episodes [15], and even suicide [16].

Consequently, the mental health of students worldwide seems to be in need of being observed and researched. This might be useful not only for the university authorities but also policy makers and governments. Since it has become evident that university restrictions will not be fully revoked in the near future, it seems necessary to research students’ well-being and subjective quality of life in order to protect their mental health and design more effective ways of handling these unique “e-times”.

Hence, the purpose of this study was to examine students’ mental health and measure the manifestation of psychopathological symptoms caused by the administrated university restrictions and overall COVID-related everyday changes. Additionally, it was designed to observe correlations between students’ subjective evaluation of these mentioned changes in academic life and their mental health. 

## 2. Materials and Methods

### 2.1. Participants

Data were collected using an online survey which was administrated between the 12th of May and the 30th of June 2020. The study was initiated by the time the academic curricula had been transferred into e-learning and most of the universities were introducing lockdowns, causing students being forced to give up their everyday life, often moving out from the closing dorms in order to move back in with their families. Students of all fields of study and faculties were invited to complete questionnaires that were submitted via e-mail addresses and social media such as Facebook. The survey was filled in by students from universities such as Wroclaw Medical University, Wroclaw University of Science and Technology, and University of Wroclaw. The survey was distributed among Polish universities via e-mail as well. Years of study and gender are listed in Table 1.

Data analyses were restricted to fully filled in forms only. All subjects gave their informed consent for inclusion before they participated in the study. The study was conducted in accordance with the Declaration of Helsinki, and the protocol was approved by the Ethics Committee of Wroclaw Medical University (signature KB 309/2020). The study was carried out by the research team of the Department of Psychiatry at the Medical University of Wroclaw. The inclusion criteria were student status and consent to participate in the study.

### 2.2. Measures

The survey consisted of four parts. The first part included sociodemographic questions concerning sex, age, place of residence and recent changes therein, employment status (number of performed jobs and any changes in this area connected with the COVID-19 outbreak), source of income, relationship status, and recent social activities. The second part focused on the general information about students’ academic career concerning their chosen field of study, mode, and year of study. The third part was designed to invite the students to evaluate changes in their academic life as administered by their universities. These were, for instance: substantive value of the online courses, their effectiveness and constitution, general distress connected with e-learning, feeling of pressure, students’ subjective productivity, and general coping strategies towards mentioned shifts in their academic routine. The last part was based on a standardized questionnaire—the General Health Questionnaire (GHQ-28, henceforth referred to as “GHQ”).

The GHQ-28 [17] is a 28-item questionnaire designed to uncover minor psychiatric disorders in the general population. It consists of four subscales: somatic symptoms (items 1,3,4,8,12,14,16), anxiety and insomnia (items 2,7,9,13,15,17,18), social dysfunction (items 5,10,11,25,26,27,28) and severe depression (items 6,19,20,21,22,23,24). Answers to the GHQ-28 are given on the 4-point Likert scale (0—not at all, 1—no more than usual, 2—rather more than usual, 3—much more than usual). The total score ranges between 0 and 84, and the higher the score, the higher the possibility of the presence of psychopathological symptoms. Commonly, a cut-off value of 24 points shows an increased risk of psychopathology [18].

### 2.3. Data Analysis

The normality of data was tested using the Shapiro–Wilk test as well as the assessment of Q-Q plots. 

Relationship analysis regarding variables obtained in author questionnaire and GHQ was performed using Mann–Whitney, Kruskal–Wallis, or Spearman correlation tests when appropriate. The effect size was obtained using the Wilcox r or eta squared. 

Given the large number of univariate analyses performed, Holm correction was applied to control for the false discovery rate. 

Analysis of independent effect that variables affected by the pandemic had on the GHQ scale was performed using multivariate regression analysis. The model was presented by reporting beta values, its 95% confidence intervals (95%CI) and *p*-values. The goodness of fit was assessed using the Akaike information criterion (AIC).

Analysis was performed in the R software for Windows (version 4.0.3) (R Core Team, 2020). Differences were considered statistically significant if the *p*-value was less than 0.05.

## 3. Results

### 3.1. Study Sample Description and Demographic Variables

A total number of 1123 participants completed the survey and GHQ. Among them, 12 participants declared their gender as “other”. As this subgroup was far too small to be comparable to the other subgroups, it was excluded from further analysis. The mean age of the studied population was 22.19 ± 2.42 years. Details describing the structure of the studied group regarding their somatic and psychiatric disorders, medication, year of study and field of study are shown in Table 1.

The mean GHQ total score was 38.84 ± 17.36, with the following mean scores in subsequent subscales: 8.76 (±4.66) in the somatic symptoms subscale, 11.40 (±5.40) in the anxiety and insomnia subscale, 11.78 ± 4.50 in the social dysfunction subscale and 7.41 ± 5.88 in the severe depression subscale. A total of 855 (76.96%) participants met the cut-off point of 24 in GHQ total score, showing increased risk for psychopathology according to previous studies. Details on general GHQ scores can be found in Table 1.

Four demographical variables were associated with the GHQ scores, though in each case the effect magnitude was considered small. Females had significantly higher GHQ total scores as well as scores of somatic symptoms, anxiety and insomnia and severe depression. Participants living in big cities (>100,000 citizens) scored lower in terms of the total GHQ score and somatic symptoms, anxiety and insomnia, and social dysfunction subscales compared to citizens of smaller cities and villages. Being forced to move back to their parents’ house due to the COVID-19 pandemic also significantly affected the GHQ scores in terms of anxiety and insomnia and social dysfunction subscale as well as the GHQ total score. Staying in a relationship significantly reduced the score only in severe depression subscale. Details regarding scores, subgroup counts, and statistical significance were shown in Tables S1–S4 (in Supplementary Material). 

### 3.2. Studies and Online Learning

There were significant negative correlations of three variables regarding online learning (value, organization, and efficiency) with the GHQ scores (all subscales and the total score). All correlations were weak or very weak (correlation coefficients above −0.39); details are presented in Table S5 (in Supplementary Materials).

Four different aspects of studying the impact on the participants were proven to create significant differences between the subgroups with small, moderate, or large effect magnitudes. Increased perceived stress due to online learning created differences in terms of GHQ somatic symptom and social dysfunction subscales and GHQ total score (moderate effect size), while the effect size on anxiety and insomnia subscale was large. Responders with increased perceived stress tended to score higher on all mentioned subscales. A change in the amount of work due to pandemic did not create significant differences in the severe depression subscale, though in terms of the rest of the subscales and the GHQ total score the differences between subgroups were significant with a small effect magnitude. In the somatic symptoms subscale, responders who reported no significant change in the amount of work scored significantly lower than the other two groups (increased or decreased amount of work), while in the social dysfunction subscale, responders who reported decreased amount of work scored the highest. Change in productivity due to pandemic created differences between subgroups in terms of GHQ total score and anxiety and insomnia subscale score with a moderate effect size, while the effect magnitude on social dysfunction subscale was large. Significantly higher scores in the somatic symptoms, anxiety and insomnia, and severe depression subscales were seen in responders who reported decreased productivity. In the case of the social dysfunction subscale, responders who reported increased productivity scored the lowest, while those who reported decreased productivity scored the highest. A large effect size was seen in the case of all subscales and the GHQ total score regarding the last important factor—thoughts about quitting studies. The presence of such thoughts created significant differences between the subgroups in all assessed subscales and the total score. Higher scores were observed in all subscales in responders who reported presence of such thoughts. Detailed data are presented in Table 2.

### 3.3. Linear Regression Analysis

In order to assess if the abovementioned variables could predict the GHQ score in all four subscales, linear regression analysis was performed. 

The somatic symptoms subscale regression model was described with the subsequent parameters: F(9,1110): 40.390, R2 = 0.248, R2 adjusted = 0.242. High values of online learning assessment were found to be a potential protective factor. On the other hand, female sex, psychotropic medication, increased perceived stress due to online learning, thoughts on quitting studies, and lower productivity due to the pandemic were associated with higher score in this subscale. Technical studies, medical studies, and greater productivity due to the pandemic could not be clearly identified either as a risk factor or a protective factor.

The anxiety and insomnia subscale regression model was described with the subsequent parameters: F(10,1099): 49.319, R2 = 0.310, R2 adjusted = 0.303. High values of online learning value assessment, living in a city of more than 100,000 citizens, and medical studies were found to be potential protective factors. Female gender, psychotropic medication, increased perceived stress due to online learning, thoughts about quitting studies, and lower productivity due to pandemic were potential risk factors. Technical studies and greater productivity due to the pandemic could not be clearly identified either as a risk factor or a protective factor.

The social dysfunction subscale regression model was described with the subsequent parameters: F(11,1098): 49.156, R2 = 0.330, R2 adjusted = 0.323. High values of online learning effectivity assessment and moving to a partner’s place of residence were found to be potential protective factors. Other changes in place of residence (but not moving to the family home), online learning value assessment, and greater productivity due to the pandemic seemed to also be protective factors, but did not reach statistical significance. Psychotropic medication, stationary studies, increased perceived stress due to online learning, thoughts about quitting studies, and lower productivity due to the pandemic were potential risk factors. Moving to the family home could not be clearly identified either as a risk factor or a protective factor.

The severe depression subscale regression model was described with the subsequent parameters: F = 12,1097: 30.478, R2 = 0.25, R2 adjusted = 0.242. High values of online learning value assessment, medical studies, and being in the third, fourth, fifth or sixth year of study were found to be potential protective factors. Female gender, psychotropic medication, increased perceived stress due to online learning, thoughts about quitting studies, and lower productivity due to the pandemic turned out to be potential risk factors. Technical studies, being on a postgraduate course, and greater productivity due to the pandemic could not be clearly identified either as a risk factor or a protective factor.

Details regarding the estimated impact of variables, statistical significance, and variables excluded from particular models are presented in Table 3. Exclusion was based on regression analysis including the Akaike information criterion.

## 4. Discussion

### 4.1. Principal Results and Comparison with Prior Findings

One of the most important findings of this study is that almost 77% of surveyed students presented with a substantial level of psychopathological symptoms, requiring psychological or psychiatric attention. Compared to the pre-pandemic literature focusing on the mental health of students, this is a larger percentage of the whole student population [19,20,21,22]. Previous studies have often used shorter versions of GHQ, e.g., GHQ-12, or the Kessler-10 (Kessler Psychological Distress Scale). Adachi et al. described in a study performed in the pre-pandemic era that a higher GHQ-12 score and the presence of somatic symptoms are important factors leading to faster searching for psychiatric or psychological consultation [20]. Considering this fact, our study points out that there is a substantially increased need for consultation access improvement for students in the era of the COVID-19 pandemic. In another pre-pandemic study, Dendle et al. found that 47.4% of respondents presented a significant level of psychological distress in Kessler-10, which is a much lower percentage than the one found in our study. GHQ-28 was also used in this study, but scoring was performed in a different manner compared to this study, so it is impossible to directly compare both results. High correlation coefficients were described for Kessler-10 and GHQ-28 scores [21]. Nevertheless, these tools are not easily comparable: Kessler-10 contains questions focused on perceived anxiety and depressive symptoms, while GHQ-28 also includes self-assessment of social functioning, insomnia, and somatic symptoms, so Kessler-10 may falsely indicate lower quantity and percentage of distressed participants. Interestingly, Dendle et al. also observed that psychological distress in long-term observation did not lead to worse academic performance [21]. Another large study from Iran, using GHQ-28, performed on female students in 2011, showed that 48.5% of participants presented an increased level of psychopathologic symptoms. Further analyses showed that birth order, marital status and family income contributed to higher GHQ scores [22]. In our study family income was not assessed, but we asked for the main financial source (employment, partner, family, scholarship, etc.) and the factor was excluded from detailed analysis as it occurred irrelevant.

Regarding previous studies on a Polish student population, significant depressive symptoms were found in 28.8% of the second year students and 14% of the fourth year students of medicine. No significant differences were found between medical and non-medical students in terms of depressive symptoms’ prevalence [23]. In another study, depressive episode prevalence across the lifespan was reported in 14.7% of students and in 9.8% when accounting for only 12 months prior to examination. Second and third year students reported depressive episodes more frequently than other students. A lack of partner was mentioned as a factor contributing to a higher prevalence of depressive episodes [24]. 

An increased frequency of psychopathological symptoms in general populations due to the COVID-19 pandemic was also reported in many countries [25,26], which shows that young student populations are generally similar to the general population with respect to psychopathological responses to the COVID-19 pandemic. In terms of student population of different fields during the pandemic, a large study from the USA showed that 48.14% of participants reported increased depressive symptoms, 38.48% reported increased anxiety, and 18.04% had suicidal thoughts [27]. PHQ-9 and GAD-7 were used in this study for the assessment of psychopathological symptoms. In another study performed among French students, 43% of participants reported significant depressive symptoms, 39.19% suffered from anxiety, and 42.94% suffered from increased distress levels [28]. In one study performed on Polish student population, 65% of responders presented with mild to severe anxiety, and 56% reported high levels of perceived stress—such high values correspond to our results. Following factors were indicated as possible confounders of increased anxiety: high stress, low general self-rated health, female gender, and frequent use of both emotion-oriented and task-oriented coping styles [10]. 

Regarding demographic variables that could determine GHQ scores, we found that female sex was associated with higher scores, while living in a big city seemed to be related to lower severity of anxiety and insomnia symptoms. Female sex has been pointed to in many papers as an important factor contributing to higher GHQ scores, especially in terms of anxiety. On the basis of a large multicentric study, Arias-de la Torre et al. reported that first-year female students present with higher level of distress, especially when they are unemployed and looking for employment (compared to those who are unemployed and not looking for a job). Poor family support and functioning level were pointed to as crucial factors contributing to higher distress level in the whole first-year student population [19]. The study was limited to first-year students and was conducted before the COVID-19 pandemic. However, in our study, employment was not a significant factor associated with psychopathology, and thus excluded from the analysis. In an already mentioned study by Ashar et al., female sex and financial insecurity also contributed to higher levels of depression, anxiety and distress, while living alone was mostly associated with more severe depressive symptoms [28].

In turn, moving to a partner’s place of residence was associated with better social functioning. However, students who moved back to the family home due to the pandemic scored higher in total GHQ and several subscales, compared to students who continued to live outside the family home. We can suppose this could be especially problematic for students whose families are not supportive, tolerant, or possibly even dysfunctional. Such observations and conclusions were presented in a paper by Gonzales et al., who performed a study in an LGBT student population, in which moving back to the family home significantly increased distress and anxiety levels in students [29]. Nevertheless, it should be mentioned that deterioration in mental health might also result from a disturbance in the process of psychosocial development itself, especially considering the fact that the young adulthood is an important life period in constituting the young person identity, building autonomy, and developing deep mutual relations with the others [30]. 

In terms of studying, we observed that medical students scored generally lower on most subscales—medical studies could contribute to less pronounced anxiety, insomnia, and depressive symptoms. This could suggest that medical students’ mental health is probably better protected against the negative impact of the COVID-19 pandemic. Xie et al. reported similar results in a large study on medical and non-medical students. Medical students presented less severe anxiety and depression symptoms, though the severity was dependent on time spent on searching for information on COVID-19 [11]. This indicates that reliable medical knowledge might be protective against negative psychopathological symptoms during pandemic. 

What is more, higher GHQ scores were present in younger students, who have just started their adult life, often changing their place of residence, and less pronounced depressive symptoms were observed in elder, “more advanced” groups of students. The first year of studying is often difficult for a young person even in non-pandemic conditions. According to Erikson’s psychosocial theory, young people face another development crisis, which is important in building a mature and integrated identity [31]. In this case, the pandemic’s consequences, such as anxiety and social isolation, pose a serious threat to young people’s adaptation skills and psychosocial resources. Other researchers also confirm these observations. Lyons et al. used the Kessler-10 test for psychopathological assessment, but the conclusion was the same—first-year students were especially at risk of developing high levels of distress. “Studying” was also the most commonly picked answer by students for a question about areas of life affected by the pandemic [1]. Similar observations were reported by Wang et al. who observed that mean GAD-7 and PHQ-9 scores were the highest in “freshmen” group and then lower in each subsequent year group [27].

Online learning seems to significantly affect students’ mental state. In the studied group, the value of online learning and the perceived stress evoked by it were significantly associated with GHQ scores. Self-reported diminished productivity was also associated with higher GHQ scores. Dendle et al. described that most stressful factors in terms of online learning were: “keeping up to date with knowledge” and “keeping up with the content to be learned”, while slightly less often mentioned factors included: “the need to do well”, “keeping up with the demands of study and examinations”, “fear of negative feedback”, and “fear of making mistakes” [21]. Finding reasons for lower satisfaction and lower value of online learning was beyond the scope of our study. The value differed significantly between different fields of studies, what also meant different universities. However, it may be hypothesized that the word “study” itself involves more than just the acquisition of knowledge. It is also connected with the sense of belonging to the university community, which is the basis of social networks. Alqurshi mentioned that main factors contributing to lower satisfaction include limited teacher–student interaction and perceived ambiguity in assignment instructions, internet connection issues, and problems with concentration in virtual classrooms. The study included both pharmaceutical students and teachers in Saudi Arabia [2]. Hasan and Bao showed in their study that fear of an academic year loss due to COVID-19 pandemic is a hypothetical mediator between the online learning “crack-up” and the presence of anxiety in students [32]. This mechanism could explain why lower value (satisfaction) assigned to online learning may contribute to worsened mental state of students also in our study. Considering factors previously described as determinants of poor online learning assessment, the need for quality improvement in terms of e-learning techniques is a novel and extremely important issue to deal with.

What we found very important is that thoughts about quitting studies were common in the studied group, and their presence was related to higher GHQ scores, which could mean that studying itself was a factor causing significant distress in students. Byrnes et al. observed that medical students reported worries that the pandemic will affect their careers, limiting possibilities of specialty choice, etc. [33].

All the data presented above suggest that the COVID-19 pandemic’s impact on students’ mental health was very significant and dramatic. However, the student population, even before the pandemic, was mentioned as an “at risk” population regarding mental health. In a robust systematic review by Ibrahim et al., it was stated that the estimated prevalence of depression equaled 30.6% among students, which is a much higher value than the prevalence in the general populations of many countries, estimated at being around 9-11%. Female sex was mentioned as an important risk factor, though the authors underlined that males are less eager to look for help from an appropriate specialist, thus both populations require more attention in terms of diagnostics and management of depression in an academic setting [34]. According to Erikson’s theory, this can be understood as a negative solution to the psychosocial crisis related to separation, building autonomy and developing identity [30,31]. Taking into account observations from the present study and the theoretical understanding of individual psychosocial development, it is necessary to approach first-year students during the pandemic with particular care. The psychopathological symptoms they are experiencing may be more than just a negative psychological response to the current pandemic. It might be the effect of the real disturbance in their psychosocial development. The researchers emphasized that so far, the university community has played an important role in this process. Adams et al. highlighted that within a university context, identity development is directly connected to the formation of psychosocial resources [31].

### 4.2. Limitations

This study has several important limitations. First of all, we assessed mostly general aspects of mental health using the GHQ questionnaire, and we did not use any tools particularly focusing on depressive or anxiety symptoms. The study had a screening and cross-sectional design without deep clinical evaluation, and thus the basis to perform cause-and-effect analysis was strongly limited. We did not include in our questionnaire thorough questions regarding family and financial situation, only general information about main income source. This also led to a limited insightfulness of questions regarding returning to family home, since reasons for this particular change having an impact on students’ well-being might, as we have noted, come from emotional burden as well as from practical reasons such as limited personal space or difficulties in accessing the Internet. The use of psychoactive substances was also not assessed in this study. 

Furthermore, we did not manage to perform detailed analysis regarding fields of studies, as some subgroups were underrepresented in our study. As the online form was easily accessible for Internet users, it was impossible to describe the target group in terms of quantity and age or sex structure. We did not manage to assess possible drop-outs and their reasons (i.e., why a responder did not fill out the questionnaire). Representativeness of the studied sample might be limited due to the lack of records concerning the number of those initially approached and reasons for some of them refusing participation. There was only one language version of the survey, written in Polish, thus we were not able to include foreign students and those who do not speak Polish. The impact of stress due to immigration was not assessed by this study, while some authors mention it as a significant factor in terms of mental state determinants in student populations [35]. Thus, our conclusions may be considered relevant only in terms of Polish users.

## 5. Conclusions

The most important conclusion of this study is that it will be important to develop recommendations for early psychosocial intervention among students and to monitor their mental health status over time. This might be performed by university counseling centers providing they exist. If not, the presented results should be particularly taken into consideration by universities’ administrations, as our results demonstrate that students could profit from interventions regarding stress reduction. This can be achieved using methods that have already proved their effectiveness among students, such as cognitive-behavioral therapy (CBT) [36,37,38], mindfulness [39] or acceptance and commitment therapy (ACT), which should be performed by mental health professionals trained in them. They can provide a new understanding of stressful situations, increase self-confidence, or empower new ways of thinking and behaving using cognitive reconstruction, behavioral activation, and challenging one’s beliefs about dealing with stress, consistent with one’s values. Moreover, these methods may be performed not only individually but also in groups [40] and online [41], which is particularly important during a crisis forcing isolation, as with the COVID-19 pandemic. All mentioned forms of therapy have already proved their effectiveness in eliminating patterns that prohibit students from undertaking adaptive coping strategies and sometimes even enhance those that are maladaptive.

One Japanese study showed that escape-avoidance coping was often adopted among healthcare providers during the COVID-19 pandemic, while excessive information seeking behaviors contributed negatively to their mental state [42]. These conclusions might be of particular use in the case of medical students, and thus we would recommend taking them into account.

In the study sample, first-year students and women were at particular risk of psychiatric distress. Moreover, university authorities should be particularly sensitive to the phenomenon of dropping out of studies during this pandemic. University authorities are facing a challenge, considering the important role of the university in the development of a young person’s identity and belonging to society. Universities should seriously consider not only how they can efficiently continue the transfer of knowledge, but also how they can continue building the student community and teacher–student relationships with the use of new technologies.

## Figures and Tables

**Table 1:** Basic demographic and health data and Global Health Questionnaire (GHQ) scores. SD—standard deviation.

Variable	*n* (%) or Mean (± SD)			
Participants	*n* = 1111			
Age (years)	22.19 (± 2.42)			
Gender	Female = 842 (75.79%)		Male = 269 (24.21%)	
Health	Psychiatric disorder = 127 (11.43%)	Somatic disorder = 151 (13.59%)	Somatic and psychiatric comorbidity = 47 (4.23%)	No chronic conditions = 786 (70.75%)
Psychopharmacotherapy	Yes = 95 (8.55%)		No = 1016 (91.45%)	
Year of study	I or II = 507 (45.63%)	III or IV = 422 (37.98%)	V or VI = 168 (15.12%)	Other (incl. postgraduate) = 14 (1.26%)
Field of study	Medical = 333 (29.97%)	Technical = 296 (26.64%)		Other = 482 (43.38%)
GHQ somatic symptoms	8.76 (±4.66)			
GHQ Anxiety and Insomnia	11.40 (±5.40)			
GHQ Social Dysfunction	11.78 (±4.50)			
GHQ Severe depression	7.41 (±5.88)			
GHQ Total	38.84 (±17.36)			
GHQ ≥ 24	*n* = 855 (76.96%)			
GHQ < 24	*n* = 256 (23.04%)			

**Table 2:** Aspects of studying and their impact on Global Health Questionnaire (GHQ) scores. SD—standard deviation.

	Increased Perceived Stress due to Online Learning			Change in the Amount of Work due to Pandemic				Change in Productivity due to Pandemic				Thoughts About Quitting Studies		
	Yes, *n* = 523; Mean (SD)	No *n* = 588; Mean (SD)	*p* Value (Effect Magnitude)	1. Did not Change, *n* = 146; Mean (SD)	2. Decreased, *n* = 373; Mean (SD)	3. Increased, *n* = 592; Mean (SD)	*p* Value (Effect Magnitude)	1. Did not Change, *n* = 199; Mean (SD)	2. Increased, *n* = 138; Mean (SD)	3. Decreased, *n* = 774; Mean (SD)	*p* Value (Effect Magnitude)	Yes, *n* = 553; Mean (SD)	No *n* = 558; Mean (SD)	*p* Value (Effect Magnitude)
GHQ somatic symptoms	10.40 (4.43)	7.29 (4.36)	<0.001 (moderate)	7.28 (4.55)	8.81 (4.52)	9.08 (4.71)	0.005 (small); 1 < 2, 1 < 3	6.93 (4.29)	7.38 (4.97)	9.47 (4.51)	<0.001 (small); 1 < 3, 2 < 3	10.57 (4.41)	6.96 (4.18)	<0.001 (large)
GHQ Anxiety and Insomnia	13.67 (4.64)	9.39 (5.23)	<0.001 (large)	9.35 (5.42)	11.24 (5.16)	12.02 (5.42)	<0.001 (small); 1 < 3, 1 < 2, 2 < 3	9.18 (5.36)	9.17 (5.69)	12.37 (5.07)	<0.001 (moderate); 1 < 3, 2 < 3	13.7 (4.64)	9.06 (5.07)	<0.001 (large)
GHQ Social Dysfunction	13.21 (4.16)	10.53 (4.41)	<0.001 (moderate)	9.69 (4.24)	12.93 (4.33)	11.59 (4.45)	<0.001 (small); 1 < 2, 1 < 3, 2 > 3	9.25 (3.88)	8.22 (4.47)	13.07 (4.01)	<0.001 (large); 1 < 3, 2 < 3, 1 < 2	13.54 (3.97)	10.04 (4.33)	<0.001 (large)
GHQ Severe depression	8.80 (6.02)	6.16 (5.48)	<0.001 (small)	6.41 (5.38)	8.01 (6.10)	7.27 (5.83)	>0.05	5.56 (5.21)	5.41 (5.42)	8.24 (5.94)	<0.001 (small); 1 < 3, 2 < 3	9.75 (5.88)	5.09 (4.89)	<0.001 (large)

**Table 3:** Regression models for all 4 Global Health Questionnaire subscales. Standard errors: Robust, type = HC1; Continuous predictors are mean-centered and scaled by 1 standard deviation. Significant *p* values are marked with bolding in the table (*p* ≤ 0.05). Variables marked with * were not included in the final model.

	GHQ Somatic Symptoms				GHQ Anxiety and Insomnia				GHQ Social Dysfunction				GHQ Severe Depression			
	Est.	2.5%	97.5%	*p*	Est.	2.5%	97.5%	*p*	Est.	2.5%	97.5%	*p*	Est.	2.5%	97.5%	*p*
(Intercept)	4.630	3.857	5.403	**0.001**	7.155	6.198	8.112	**0.001**	7.304	6.379	8.229	**0.001**	4.399	3.341	5.458	**0.001**
Female gender	1.305	0.771	1.839	**0.001**	1.354	0.749	1.960	**0.001**	*	*	*	*	0.844	0.121	1.567	**0.02**
City > 100,000 citizens	*	*	*	*	−0.740	−1.277	−0.204	**0.007**	*	*	*	*	*	*	*	*
Change in residence—other	*	*	*	*	*	*	*	*	−0.976	−2.015	0.057	0.06	*	*	*	*
Change in residence—moved to partner	*	*	*	*	*	*	*	*	−1.567	−2.599	−0.536	**0.003**	*	*	*	*
Change in residence—moved to family home	*	*	*	*	*	*	*	*	0.265	−0.203	0.733	0.27	*	*	*	*
Stationary studies	*	*	*	*	*	*	*	*	1.425	0.579	2.271	**0.001**	*	*	*	*
Medical studies	−0.477	−1.070	0.116	0.12	−1.030	−1.707	−0.354	**0.003**	*	*	*	*	−1.121	−1.858	−0.384	**0.003**
Technical studies	0.144	−0.439	0.726	0.63	−0.115	−0.759	0.529	0.73	*	*	*	*	−0.382	−1.145	0.381	0.33
Year of study—III or IV	*	*	*	*	*	*	*	*	*	*	*	*	−0.975	−1.667	−0.282	**0.006**
Year of study—other (incl. postgraduate)	*	*	*	*	*	*	*	*	*	*	*	*	−0.557	−3.475	2.362	0.71
Year of study—V or VI	*	*	*	*	*	*	*	*	*	*	*	*	−0.964	−1.877	−0.051	**0.04**
On psychotropic treatment	1.983	1.024	2.942	**0.001**	1.015	0.009	2.022	**0.048**	0.938	0.117	1.760	**0.03**	4.541	3.464	5.618	**0.001**
Online learning—value	−0.361	−0.613	−0.110	**0.005**	−0.326	−0.617	−0.035	**0.03**	−0.203	−0.486	0.080	0.16	−0.541	−0.877	−0.205	**0.002**
Online learning—effectivity	*	*	*	*	*	*	*	*	−0.651	−0.953	−0.349	**0.001**	*	*	*	*
Increased perceived stress due to online learning	2.045	1.524	2.565	**0.001**	2.830	2.245	3.416	**0.001**	0.907	0.430	1.385	**0.001**	1.054	0.393	1.716	**0.002**
Thoughts on quitting studies	2.581	2.042	3.121	**0.001**	3.245	2.631	3.859	**0.001**	1.954	1.476	2.432	**0.001**	3.613	2.914	4.311	**0.001**
Change in productivity due to pandemic—faster	0.624	−0.291	1.538	0.18	0.237	−0.823	1.297	0.66	−0.697	−1.566	0.171	0.12	−0.023	−1.036	0.990	0.96
Change in productivity due to pandemic—slower	1.074	0.451	1.697	**0.001**	1.274	0.538	2.011	**0.001**	2.499	1.877	3.121	**0.001**	0.934	0.149	1.719	**0.02**

## Data Availability

Data is contained in this article and provided supplementary material. On demand additional data can be obtained from the corresponding author, they are not publicly available due to privacy restrictions.

## Supplementary Materials

The following are available online at https://www.mdpi.com/1660-4601/18/6/2884/s1, Table S1: Gender differences in Global Health Questionnaire (GHQ) scores. SD—standard deviation, Table S2: Living place differences in Global Health Questionnaire (GHQ) scores. SD—standard deviation, Table S3: Change of residence due to pandemic—impact on Global Health Questionnaire (GHQ) scores. SD—standard deviation, Table S4: Relationship impact on Global Health Questionnaire (GHQ) scores. QR—quartile range, SD—standard deviation, Table S5: Spearman’s R correlation coefficients and testing *p* values for three online learning assessment criteria and Global Health Questionnaire (GHQ) scores.
