# Supplementary material for: Class of 2020 in Poland: Students’ Mental Health during the COVID-19 Outbreak in an Academic Setting
Source: Int J Environ Res Public Health. 2021 Mar 11;18(6):2884. doi: 10.3390/ijerph18062884 (PMC7999098; doi:10.3390/ijerph18062884)
Supplement: Supplementary file 1 [file ijerph-18-02884-s001.pdf]

Table S1. Gender differences in Global Health Questionnaire (GHQ) scores. SD—standard deviation.

	**Female (*n* = 842)**		**Male (*n* = 269)**		***p***
	**Mean**	**SD**	**Mean**	**SD**	
GHQ Somatic Symptoms	9.06	4.72	7.81	4.33	0.001
GHQ Anxiety and Insomnia	11.71	5.39	10.43	5.32	0.002
GHQ Social Dysfunction	11.76	4.51	11.86	4.48	0.67
GHQ Severe Depression	7.64	5.86	6.70	5.90	0.009
GHQ Total	39.65	17.40	36.32	17.04	0.009

Table S2. Living place differences in Global Health Questionnaire (GHQ) scores. SD—standard deviation.

	**Big City > 100,000 (*n* = 580)**		**Small City or Village < 100,000 (*n* = 531)**		***p***
	**Mean**	**SD**	**Mean**	**SD**	
GHQ Somatic Symptoms	8.46	4.64	9.08	4.65	0.021
GHQ Anxiety and Insomnia	10.82	5.48	12.04	5.25	0.001
GHQ Social Dysfunction	11.35	4.51	12.26	4.45	0.001
GHQ Severe Depression	7.21	5.86	7.63	5.91	0.2
GHQ Total	37.32	17.17	40.50	17.44	0.003

Table S3. Change of residence due to pandemic—impact on Global Health Questionnaire (GHQ) scores. SD—standard deviation.

	**No Change of Residence (*n* = 471)**		**Moved to Family House (*n* = 551)**		***p***
	**Mean**	**SD**	**Mean**	**SD**	
GHQ somatic symptoms	8.32	4.56	9.09	4.70	0.08
GHQ Anxiety and Insomnia	10.79	5.43	11.99	5.26	0.003
GHQ Social Dysfunction	11.38	4.56	12.34	4.41	0.002
GHQ Severe depression	7.06	5.85	7.75	5.86	>0.05
GHQ Total	37.04	17.26	40.66	17.19	0.005

Table S4. Relationship impact on Global Health Questionnaire (GHQ) scores. QR—quartile range, SD—standard deviation.

	**No Partner (*n* = 528)**		**Has a Partner (*n* = 558)**		***p***
	**Mean**	**SD**	**Mean**	**SD**	
GHQ somatic symptoms	8.67	4.66	8.91	4.63	>0.05
GHQ Anxiety and Insomnia	11.31	5.28	11.59	5.46	>0.05
GHQ Social Dysfunction	12.14	4.48	11.50	4.47	>0.05
GHQ Severe depression	7.94	6.06	7.07	5.69	0.03
GHQ Total	39.58	17.20	38.53	17.34	>0.05

Table S5. Spearman's R correlation coefficients and testing *p* values for three online learning assessment criteria and Global Health Questionnaire (GHQ) scores.

	**Online Learning Value**		**Online Learning Organization**		**Online Learning Effectivity**	
	**Spearman R**	***p* value**	**Spearman R**	***p* value**	**Spearman R**	***p* value**
GHQ somatic symptoms	−0.19	<0.001	−0.12	0.002	−0.2	<0.001
GHQ Anxiety and Insomnia	−0.2	<0.001	−0.13	0.002	−0.24	<0.001
GHQ Social Dysfunction	−0.27	<0.001	−0.17	<0.001	−0.36	<0.001
GHQ Severe depression	−0.2	<0.001	−0.16	<0.001	−0.22	<0.001
GHQ Total	−0.25	<0.001	−0.17	<0.001	−0.29	<0.001
